# Contributions to Pathology and the Practice of Medicine

**Published:** 1885-12

**Authors:** 


*Contributions to Pathology and the Practice of Medicine.* By J. R. WARDELL, M.D. Pp. 807. London: H. R. LEWIS. 1885.

By the advice of friends who stand high in the profession, Dr. Wardell has collected in this large volume the sum of his contributions to scientific medicine. Many of the papers are classics, and have long passed beyond the criticism of the reviewer: among these, we would specially call attention to the article on "Relapsing Fever," which still remains the best in existence. Of the others—and they are very numerous and very varied—nearly all have appeared at different periods in journals or other medical publications. Some of these are of a very high order of excellence; there is not one that is commonplace or mediocre.

Dr. Wardell's death has followed quickly on the fruition of his labours. He leaves behind him a valuable legacy in this work, the result of many years of quiet, honest labour, the outcome of a scientific and cultured mind.

